# Effectiveness of dialectic behavioral therapy in routine outpatient care: the Berlin Borderline Study

**DOI:** 10.1186/2051-6673-1-20

**Published:** 2014-12-18

**Authors:** Christian Stiglmayr, Julia Stecher-Mohr, Till Wagner, Jeannette Meiβner, Doreen Spretz, Christiane Steffens, Stefan Roepke, Thomas Fydrich, Harriet Salbach-Andrae, Julian Schulze, Babette Renneberg

**Affiliations:** Arbeitsgemeinschaft fuer Wissenschaftliche Psychotherapie, Witzlebenstraβe 30a, 14057 Berlin, Germany; Department of Psychology, Humboldt-Universitaet zu Berlin, Unter den Linden 6, 10999 Berlin, Germany; Department of Psychology, Freie Universitaet Berlin, Habelschwerdter Allee 45, 14195 Berlin, Germany; Department of Psychiatry, Charité, Universitaetsmedizin Berlin, Campus Benjamin Franklin, Eschenallee 3, 14050 Berlin, Germany; Department of Psychiatry, Charité, Universitaetsmedizin Berlin, Campus Virchow-Klinikum, Augustenburger Platz 1, 13353 Berlin, Germany

**Keywords:** Borderline personality disorder, Dialectical behavior therapy, Effectiveness study

## Abstract

**Background:**

Dialectical behavior therapy (DBT) has been proven to be an efficacious treatment for borderline personality disorder (BPD) in several randomized controlled trials (RCTs). However, generalizability of this outcome to the routine health care (effectiveness) has rarely been investigated to date. The aim of this study is to examine the effectiveness of DBT for BPD under the routine health care situation in Germany.

**Methods:**

The study has a longitudinal design over a course of four years with six assessment points. In this paper, results for the first year of treatment are reported. Outcome was assessed at four times throughout an initial phase (of up to five therapy-sessions) and an additional 12 months of therapy. Overall, *n* =78 patients started the study, 47 patients completed one year of treatment. Dependent variables were number and duration of inpatient treatment stays, number of suicide attempts and non-suicidal self-injury, severity of borderline symptoms, depression, level of dissociation, and general psychopathology.

**Results:**

Patients significantly improved regarding self-injurious behaviors, number of inpatient hospital stays, severity of borderline symptoms and psychopathology. At the end of the first treatment year, 77% of the patients no longer met criteria for BPD diagnosis. Fewer therapy discontinuations by patients were observed when therapists participated in consultation teams.

**Conclusions:**

Under routine mental health care conditions in Germany, outpatient DBT leads to positive results comparable to those reported in other effectiveness studies and in randomized controlled trials.

## Background

A number of randomized controlled trials (RCTs) have demonstrated the efficacy of outpatient dialectical behavior therapy (DBT) [1] for the treatment of patients with borderline personality disorder (BPD) [2–11]. Five of these studies compared DBT with treatment-as-usual (TAU) [2–6], one study with a client-centered approach [7], one study with TFP and supportive therapy [10] and two other studies with treatment by experts [8, 9]. Except for the studies by Feigenbaum et al. [2], McMain et al. [9], and Clarkin et al. [10], only female participants were included in these trials. In the study by Feigenbaum et al. [2] patients with BPD as well as patients with another Cluster B personality disorder were included, with the majority (92%) meeting BPD criteria. In these studies, length of treatment varied between 6 and 12 months. In their meta-analysis, Stoffers et al. [11] conclude that DBT is the only approach developed for the treatment of borderline patients with several RCT comparison studies available. Thus, evidence-based treatment guidelines including those from the American Psychological Association and the German S2-Guidelines assign the highest empirical evidence for DBT as a treatment approach for BPD [12, 13].

DBT has proven especially effective in reducing self-injuriousbehavior, suicide attempts and inpatient treatment days. It should also be noted that treatment with DBT showed a marked reduction of disorder-related direct and indirect monetary costs [14–17].

For several reasons, generalizability of RCTs to the actual routine health care situation is limited. While efficacy-studies – usually with high internal validity - answer the question whether a specific intervention works for a specific clinical problem, effectiveness aim to investigate how a certain treatment approach works under clinical routine circumstances and thus enlarges the external validity.

In order to transfer results of RCT studies to routine clinical care, a three-phased program for the evaluation of psychotherapy has been proposed: a pilot phase (stage 1) followed by RCTs with the aim to investigate the efficacy of a treatment form (stage 2) and the conduction of effectiveness studies under routine clinical settings (stage 3) e.g. [18]. Accordingly, the necessity of effectiveness studies has also been emphasized for the treatment of borderline patients, e.g. [19–22]. Several studies were conducted investigating effectiveness of DBT under routine health care conditions [23–27]. Results indicate high effectiveness of the treatment, mostly based on report of pre-post changes of relevant outcome variables. So far, a study by Friedrich and colleagues [25] is the sole German effectiveness-study showing positive outcome for outpatient DBT. Conclusions from this study are limited, however, because only patients whose health insurance covered an unusually high number of individual therapy sessions and skills group sessions were included in this trial. Therapy with borderline patients is regarded as particularly challenging for therapists [28–30]. Therefore, weekly consultation team meetings are an essential part of DBT. Besides the prevention of burnout, the additional goal of consultation team meetings is to ensure DBT treatment adherence. To our knowledge, the impact of concurrent supervision and consultation team meetings on therapy course and outcome has not yet been explored.

The aim of the current study was to investigate the effectiveness of DBT with BPD patients under routine mental health care conditions in Germany. In Germany, DBT is mostly offered within existing networks of therapists and institutions. Our study was conducted within the Berlin borderline network and thus also serves as an evaluation of such a network. Based on other publications on the effectiveness of DBT, we expected positive outcome for the following parameters: number and duration of inpatient stays, frequency of suicide attempts, frequency of self-injurious behaviors, extent of borderline symptomatology and reduction of general and specific psychopathology. In addition, we explored whether participation of therapists in concurrent supervision and continuous attendance of consultation teams were related to premature termination of therapy.

## Methods

### Study design and procedure

The current study has a longitudinal design with a total of six assessment times over a period of four years. Here, results for the first treatment year with four assessment times are reported. Within the German health care system five initial sessions of psychotherapy are always covered by health insurances. This initial phase was added to the one year of therapy. The treatment was provided by psychotherapists in private practice, all trained in DBT (more information on therapists is provided below).

Subsequent to a telephone screening, possible participants were invited to participate in an extensive diagnostic assessment procedure (t0). A team of trained master-level psychologists conducted the telephone screening as well as the diagnostic procedure. All participants gave informed consent for participation. The study was approved by the ethics committee of Charité Berlin.

Patients who met inclusion criteria (see below) were referred to one of the participating therapists. Additional assessment points were: (t1) after the initial phase of treatment (first five sessions), and at 4 months (t2) and 12 months after t1 (t3). Follow-up assessments at 24 and 48 months are planned but are not part of the presented analyses. Patients had the opportunity to continue with DBT after t3.

The median number of days between enrolment (t0) and t1 was 120 days. This time included the application process for coverage of treatment costs by the health insurance as well as the first 5 therapy sessions. In routine psychotherapeutic care in Germany this process takes about 2-3 months. The rather large range was due to limited capacities of the therapists, problems in finding an appointment for the first therapy session or to patients not showing up at the arranged dates. The initial phase (five sessions; see above) was held prior to assessment of t1.

### Participants

Patients were recruited through a network of in- and outpatient clinicians and institutions as well as training programs and community mental health centers. When interested, patients received a note including a short description of the study and the telephone number of the study center. Furthermore, a homepage referred to the study (http://www.borderline-netzwerk-berlin.de).

Inclusion criteria were a current diagnosis of borderline personality disorder (BPD) according to DSM-IV-TR [31] as assessed by SCID-II [32] and a minimum age of 16 years. Exclusion criteria were lifetime diagnoses of schizophrenia, bipolar I disorder, acute suicidality, substance dependence within the last six months, a body-mass-index lower than 18 and an IQ lower than 80 as well as presence of a diagnosis of antisocial personality disorder. Additionally, participants were not included when they were in an ongoing psychotherapy.

**Figure 1:**
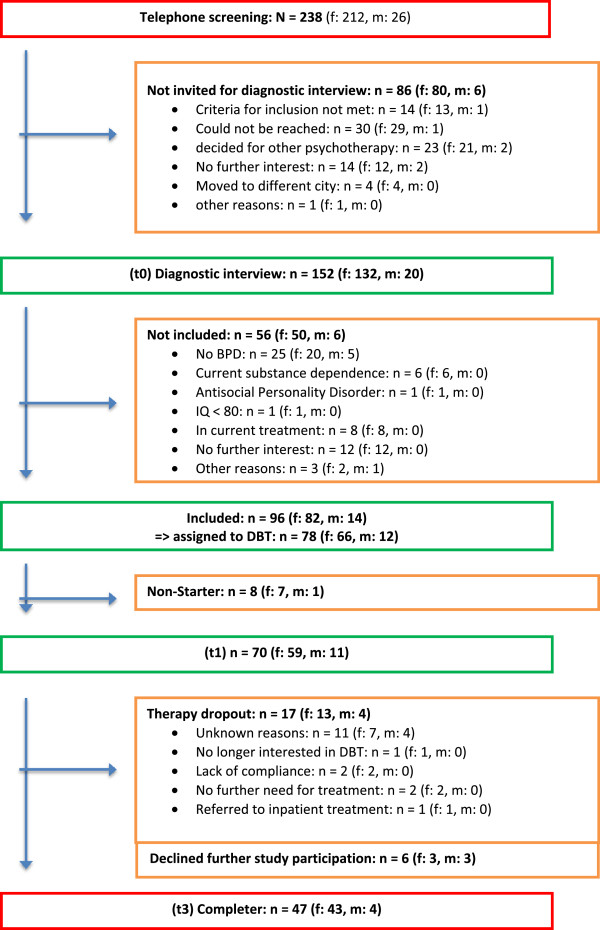
**Participant Flow.**

*N* =238 individuals interested in participation were screened by phone (see Figure 1). 152 patients were invited for further assessment; 56 did not meet inclusion criteria. Due to the limited capacities of available DBT therapists, 18 patients meeting inclusion criteria were not treated within a DBT protocol but were rather referred to other CBT therapists. Those patients did not differ significantly in relevant characteristics (age, gender, severity of borderline symptoms) from the DBT patients. As our aim was to focus on treatment outcome for DBT in a naturalistic setting, data of these 18 patients were not included in this report. Of the remaining *n* =78 patients who completed the diagnostic procedure (t0) eight did not start treatment (non-starter); thus 70 patients (59 female) started DBT treatment. Seventeen patients dropped out of treatment. Following the DBT rules within the Berlin borderline network, patients were considered dropouts when they missed four or more consecutive scheduled sessions. Further six patients did not provide data at 12-month assessment time and discontinued study participation. Therefore, *n* =47 patients (43 female) completed the one-year treatment and provided data (see Figure 1).

Table 1 shows sociodemographic as well as clinical variables of the *n* =47 participants at t0. Participants had a mean age of 30.1 years (*SD* =8.1). Four of the 47 participants were male (8.5%). The mean number of diagnostic criteria met for BPD was 6.5 (*SD* =1.2). On average, patients had more than two additional Axis I disorders (range 0–7) and more than one additional personality disorder (range 0–4). The median number of psychotropic medications was 2 (range 0–7).

**Table 1:** **Sociodemographic and clinical characteristics of patients**

	Completer	
	***n*** = 47	
	***M***	***SD***
Age	30.1	8.1
male	30.8	7.1
female	30.0	8.3
	***n***	***%***
Gender		
male	4	8.5
female	43	91.5
Education		
No formal education	1	2.1
9 years	4	8.5
10 years	28	59.6
High-school	14	29.8
Family status		
Single	18	38.3
Married or in a steady relationship	29	61.7
Occupational status		
Retired due to medical reasons	9	19.1
unemployed	21	44.7
student	8	17.0
Working/employed	9	19.1
Any psychotropic medication	33	70.2
Antidepressants	25	53.2
Neuroleptics	13	27.7
Benzodiazepines	4	8.5
Other psychotropic medication	24	51.1
Comorbid disorders (current)		
Any depressive disorder	18	38.3
Panic disorder w agrophobia	7	14.9
Social phobia	11	23.4
Obsessive compulsive disorder	3	6.4
PTSD	17	36.2
Other anxiety disorder	11	23.4
Any anxiety disorder	29	61.7
Eating disorder	17	36.3
Substance abuse	12	25.5
Any Axis-I disorder	43	91.5
Other personality disorder	32	68.1

### Treatment and therapists

Treatment was offered within the already existing network of therapists and institutions, the Berlin borderline network. In line with the recommendations by Linehan [1], DBT within this network consisted of a weekly individual therapy session (50 min) and a weekly skills group training session (120 min). In addition, treatment elements included telephone contacts between the individual therapist and the patient, foremost for crisis intervention, as well as a consultation team meeting (60 min) at least once every week for up to five individual therapists in which treatment for the patients was discussed and planned. Supervision was offered throughout to all therapists and was conducted by a certified DBT supervisor (CS); participation was voluntary.

The mean number of therapy sessions was 38.5 (*n* =47; *SD* =9.2; range 17–55). As part of the study, six DBT skills groups were offered. Of the 47 patients at t3, 42 (89.4%) attended the skills training over an average of 18.9 sessions (*SD* =10.0; min =12 max =36).

DBT therapists were recruited via the Berlin borderline network. Therapists had attended at least 64 hours of DBT training at an institute for DBT training certified by Linehan. A requirement for the skills trainers was that one of them had conducted all skills training modules at least once before. All individual therapists had a medical or psychological background and were required to have the psychotherapy state license (German: Approbation). All worked in private practice and provided treatment as part of the routine mental health care in Germany.

Treatment of the 47 patients (in square brackets information is given for the 70 patients who were treated with DBT) was provided by 20 therapists (13 female; mean age 41.2 years, *SD* =6.6) [29 therapists; 22 female; mean age 40.9 years, *SD* =6.7]. They had an average of 13 years (*SD* =6.1) [12.6 years, *SD* =5.7] of professional experience as psychotherapists and had been providing DBT on average for 5.9 years (*SD* =3.4) [5.7 years, *SD* =3.8]. The mean number of DBT training classes attended was 5.9 (*SD* =1.4) [5.5, *SD* =1.7]. Each therapist treated on average 3.3 patients (*SD* =1.8; range 1–7) [2.8 patients; *SD* =1.8, range 1–7]. Four [also 4 therapists for the 70 patients] therapists were certified trainers in DBT, three [also 3 therapists for the 70 patients] were certified DBT supervisors. Seventeen therapists (85%) [22 therapists, 75.6%] attended a weekly consultation team, 16 (80%) [16 therapists, 55.2%] regular DBT supervision.

### Assessment and measures

At t0 the German versions of the SCID I [33] and SCID II [32] for DSM-IV were applied. Diagnosticians were master-level clinical psychologists with a completed or undergoing CBT training program, had a mean of 9.5 years of clinical experience and had completed at least 22 hours of training with co-authors T.F. and B.R. for the SCID interviews. Diagnostic assessments were supervised by B.R. Interrater reliability (intraclass correlation coefficient) for a diagnosis of BPD diagnosis was excellent (ICC =0.96). Estimation of patients’ level of intelligence was based on word fluency vocabulary test (WST; [34]).

To determine the number of suicide attempts and non-suicidal self-injury (NSSI), the Lifetime Parasuicide Count (LPC; [35]) was conducted; number and length of inpatient or partial inpatient stays were assessed with a report tool developed by Wagner et al. [16, 17]. To assess severity of the borderline symptoms, the Borderline Symptom List (BSL; [36]) and the borderline section of SCID-II were performed at t3 again. Borderline-specific thinking patterns were assessed by the Questionnaire of Thoughts and Feelings (QTF; [37, 38]). Further, the Brief Symptom Inventory (BSI; [39]) was applied to assess general psychopathology. For depression the Beck-Depression-Inventory (BDI; [40]) and the Hamilton-Depression-Scale were applied (HAM-D; [41]); the level of dissociative symptoms was assessed with the Dissociation-Tension-Scale (DSS; [42]).

Therapists’ adherence to DBT was rated with the Adherence Coding Scale [43]. The scale comprises 66 items and allows differentiated rating of the extent and the competence of the application of the various DBT strategies on the part of the therapists. For each item a score between 0 and 5 was assigned, scores ≥4 indicate the adherent application of a strategy. Prior to the study, three raters were extensively trained by Katie Korslund, Behavioral Research & Therapy Clinics (BRTC), until a satisfactory calibration was achieved. In the first three months of the treatment, two randomly selected video-taped therapy sessions were rated for adherence, in the following three-month periods one video-taped session. In total, a maximum of five video tapes per therapy were rated for adherence. The mean value for all rated sessions was 4.17 (only completers; *n* =43; *SD* =0.12; range 3.78–4.41) indicating an adherent application of DBT.

### Statistical procedures

In order to analyze the short- and long-term effects of DBT, a multilevel analysis was conducted on outcome variables. A dummy variable coding scheme was used to identify the three time periods under investigation: t0 to t1 (pre-assessment until start of therapy), t1 to t2 (short-term effect after 4 months) and t1 to t3 (long-term effect after 12 months). Assessment time t1 served as a reference category in relation to t0, t2, and t3. Dummy variables were integrated into the multilevel model on level 1. On level 2, the coefficient  describes the mean value of the dependent variable at the onset of therapy, the coefficient the mean change of the symptoms from t0 to t1 (pre-assessment until beginning of therapy) and so on. Assuming that changes in symptoms vary between participants, the random effects of the difference scores were allowed for. It is recommended to keep the random effects at a maximum as long as there are no convergence problems (e.g. [44]).

The equation for the multilevel was:


For the following dependent variables the multilevel model was computed: BDI, BSI-GSI, BSL, DSS, QTF, HAM-D. As there was considerable variation over the time period from pre-assessment (t0) to t1 after the first five sessions, it was additionally controlled whether this initial period offered a significant explanatory contribution for the variability on the mean change. However, as in none of the models a significant influence was observed, this assessment period was not included in the computation model.

In addition to the completer analysis, the same multilevel model was applied for the intention-to treat (ITT) sample (*n* =78). Note that *n* varies across outcome measures and time points.

Besides statistical significance, effect size estimates according to Cohen [45] (for dependent data: dz = |μz |/σz = |μx − μy|/√(σx2 + σy2 − 2ρxy σx σy)) were computed. The effect size estimates are based on the pair-wise consideration of the assessments at t0 and t3.

Because of the skewed distribution of the number of suicidal behaviors and self-injurious behavior as well as inpatient days, it was not possible to conduct multilevel analyses for these variables. These variables were evaluated using non-parametric procedures. The impact of confounding variables was controlled for with covariate analyses. Results were double-checked non-parametrically with the Wilcoxon-Test and Friedman’s analysis of variance by ranks. Correlations were computed with Pearson’s *r*, Spearman’s rho or point biserial correlations. Comparisons of frequency of a characteristic between two independent samples were controlled for with the Fisher-Yates test, for dependent variables with the McNemar test. Nominal data were calculated by using X^2-^test.

## Results

No significant difference between completers (*n* =47), non-completers (*n* =23) and the ITT sample (*n* =78) were observed regarding relevant variables (age, sex, education, number of psychotropic medication, number of DSM-IV-TR BPD criteria as well as the outcome variables of borderline symptomatology and psychopathology).

As shown in Table 2, significant reductions in non-suicidal self-injury (NSSI), number and duration of inpatient treatment stays, as well as in number of diagnostic criteria met for BPD were observed.

**Table 2:** **Median, range, descriptive means (** ***M***_***d***_ **) and standard deviations (** ***SD*** **) for pair-wise comparisons t0–t3**

	t0		t3			Time effect			
	Median	Range	Median	Range	***n*** (pairs)		Wilcoxon ***U***	***p***	Effect size ^d^
Number of suicide attempts	.00	0-2	.00	0-1	42			1.000^a^	
NSSI	5.17	0-901	1.00	0-174	42		-3.03	.002	0.33
	***M***_***d***_	***SD***	***M***_***d***_	***SD***	***n*** **(pairs)**	***t***	***df***	***p***	**Effect size** ^**d**^
Inpatient treatment									
Number of inpatient stays	1.13	1.41	0.32	0.89	47	3.85	46	<.001	0.56
Duration (days)	51.3	74.2	6.8	19.9	47	4.15	46	<.001	0.61
Number of DSM-IV TR BPD criteria met	6.4	1.2	3.2	1.9	31	8.85	30	<.001	1.59

Note. Effect sizes (Cohen’s *d*) are based on pair-wise descriptive statistics t0-t3 (*p*-values pair-wise Wilcoxon- and *t*-tests).

Number of suicide attempts, non-suicidal self-injury, number and days of inpatient treatment stays 12 months prior t0 is compared to 12 months prior t3 NSSI.

^a^McNemar-Test because of dichotomous variables (suicide attempt yes/no).

### Suicide attempts

Six patients reported one or more suicide attempts within the 12 months prior to the study; in the course of the one-year treatment period only one patient reported a suicide attempt. This participant was not one of the 6 aforementioned patients. Since most of the participants reported no suicide attempt (mode =0); the difference from t0 to t3 regarding suicide attempts was not statistically significant (Table 2).

### NSSI

Patients showed a decrease in NSSI over time with a small effect size (*d* = .33). At time of study inclusion, 15 participants reported no NSSI (32%) in the past 12 months. A total of 11 patients did not show NSSI prior to or over the course of the data collection period (26%; *n* =42). Three persons who did not report any NSSI prior to treatment (t0) showed NSSI during the treatment period. Due to the skewed data distribution, NSSI was tested non-parametrically.

### DSM-IV TR BPD criteria

Due to missing data, SCID-II ratings for BPD features were obtained only for *n* =31 participants at t3. Of those 31 patients, *n* =24 (77%) did no longer meet DSM-criteria for BPD.

Neither gender nor education nor age of the patients had a significant impact on the change in the dependent variables between t0 and t3. Only for the number of medications at study inclusion a positive correlation with the changes during the one-year DBT treatment was found with the number of days spent in inpatient treatment (*r* = .33, *p* <0.05). Patients with a higher number of medications showed a larger reduction in the number of inpatient hospital days during outpatient DBT treatment. Therefore, this variable was controlled for when appropriate.

### Borderline symptoms and psychopathology

Table 3 shows results for self-reported severity of borderline symptoms, depression, dissociation and overall symptom severity for all assessment times. Results indicate significant improvement on all measures with medium effect sizes between *d* =0.43 and *d* =0.66.

**Table 3:** **Descriptive means (** ***M***_***d***_ **) and standard deviations (** ***SD*** **) for pair-wise comparisons t0–t3**

	t0		t3			Time effect			
	***M***_***d***_	***SD***	***M***_***d***_	***SD***	***n*** (pairs)	***t***	***df***	***p***	Effect size ^d^
BSL	2.13	0.55	1.69	0.88	37	2.97	36	.005	0.49
QTF	3.69	0.53	3.32	0.75	34	3.08	33	.004	0.53
BDI	30.9	8.6	22.4	14.3	39	4.10	38	<.001	0.66
HAM-D	11.68	4.74	9.06	6.50	31	2.40	30	.023	0.43
BSI-GSI	1.91	0.65	1.52	0.91	39	2.69	38	.010	0.43
DSS	28.5	16.2	19.6	13.9	31	3.39	30	.002	0.61

Note. Effect sizes (Cohen’s *d*) are based on pair-wise descriptive statistics t0-t3 (*p*-values obtained from pair-wise t-tests).

BSL= Borderline Symptom List.

QTF = Questionnaire of Thoughts and Feelings borderline-specific cognitions.

BDI = Beck Depression Inventory.

HAM-D = Hamilton Depression Scale.

BSI-GSI = Brief Symptom Inventory – Global Severity Index.

DSS = Dissociation-Tension-Scale.

As shown in Table 4, the multi-level-model implied that mean values are very close to the descriptive statistics per assessment time, indicating a good fit of the model to the data.

**Table 4:** **Borderline symptoms and psychopathology: descriptive means (** ***M***_***d***_ **), standard deviations (** ***SD*** **) and multilevel-model implied means (** ***M***_***m***_ **)**

	***M***_***d***_ and ***SD***				***M***_***m***_				***Difference scores***		
Outcome	t0	t1	t2	t3	t0	t1	t2	t3	t0-t1	t1-t2	t1-t3
BSL	2.10 (0.54)	1.89 (0.73)	1.81 (0.74)	1.68 (0.89)	2.09	1.90	1.87	1.70	-0.19*	-0.03	-0.19
QTF	3.73 (0.55)	3.62 (0.55)	3.42 (0.71)	3.25 (0.82)	3.71	3.62	3.49	3.25	-0.10	-0.13	-0.36***
BDI	31.12 (8.60)	26.78 (12.24)	24.27 (10.22)	21.72 (13.97)	30.77	27.07	24.89	22.21	-3.70*	-2.18	-4.87*
HAMD	11.69 (4.67)	11.09 (6.31)	7.42 (4.60)	8.04 (6.11)	12.13	10.81	8.17	9.09	-1.32	-2.64***	-1.71*
BSI-GSI	1.92 (0.64)	1.95 (0.73)	1.64 (0.80)	1.46 (0.90)	1.92	1.93	1.67	1.49	0.01	-0.26*	-0.44***
DSS	29.70 (16.35)	25.95 (19.08)	22.27 (17.26)	20.03 (16.84)	30.18	26.16	23.90	20.64	-4.02*	-2.26	-5.52**

*Note:* *** *p* < .001; ** *p* < .01; * *p* < .05.

*P*-values are based on multilevel model difference scores *β*_10_(t0-t1), *β*_20_ (t1-t2), *β*_30_ (t1-t3) (*n* = 47).

Change in symptoms between different assessment points was analysed, and results are reported in Table 4. From the diagnostic pre-assessment (t0) to the assessment point after the first five sessions (t1), significant changes occurred in self-reported depression (BDI), dissociative symptoms (DSS), and in borderline symptoms (BSL). Regarding the short-term effect (t1–t2) significant differences for the BSI-GSI as well as for the HAM-D were observed. For the period from t1 to t3 there was a significant reduction in symptoms of depression (BDI and HAM-D), global severity of symptoms (BSI-GSI), dissociation (DSS) and also in borderline-specific cognitions (QTF).

The intention-to-treat analysis including *n* =78 patients showed no substantial differences on any of these outcome variables. Equally important, there were no differences in the interpretation of the *p*-values at the 5% level with two exceptions: The t0-t1 difference score for the BSL was not significant and the t1-t3 difference score was also not significant in the ITT sample.

In addition, reliable change and clinical significant change were calculated following Jacobson et al. [46]. Depending on the outcome measure, between 35.5% and 38.5% of the participants can be considered recovered or reliably improved in borderline symptoms and psychopathology (Table 5).

**Table 5:** **Reliable change and clinical significant change on outcome measures**

	BPD baseline		NCS**			Recovered		Improved*		Unchanged		Deteriorated	
Outcome	***M***	***SD***	***M***	***SD***	***CRIT***	***n***	%	***n***	%	***n***	%	***n***	%
BDI (*n* = 39)	30,85	8,60	7,72	6,47	17,65	10	25.6	14	35.9	23	59.0	2	5.1
BSI-GSI (*n* = 39)	1,91	0,65	0,31	0,23	0,73	5	12.8	15	38.5	18	46.2	6	15.4
BSL (*n* = 37)	2,13	0,55	0,40	0,22	0,89	6	16.2	14	37.8	19	51.4	4	10.8
DSS (*n* = 31)	28,47	16,20	2,79	2,90	6,69	2	6.5	11	35.5	19	61.3	1	3.2
FGG (*n* = 34)	3,69	0,53	1,99	0,53	2,84	4	11.8	13	38.2	17	50.0	4	11.8
HAMD (*n* = 31)	11,68	4,74	0,91	1,04	2,85	2	6.5	11	35.5	17	54.8	3	9.7

*Note: improved category contains both improved and recovered patients.

BPD: Borderline Personality Disorder *M/SD* at baseline (t0).

NCS: non-clinical samples *M/SD*.

**Descriptives derived from: BDI [46], BSI-GSI [40], BSL [47], DSS [41], QTF [37], HAMD [48].

### Attendance of a concurrent supervision or consultation team

When therapists attended the consultation team meetings, significantly fewer patients dropped out of treatment (X^2^ = 8.05; *p* < .05). No significant effect was observed for the influence of attended supervision on the rate of dropouts (supervision yes/no: *r*_bis_ = -.23; *p* < .05; number of supervision sessions: *r*_bis_ = -.45; *p* < .01).

## Discussion

The present study examined the effectiveness of outpatient DBT under routine mental health care conditions in Germany. As far as we know this is the second effectiveness study ever performed in Germany regarding outpatient DBT.

In line with results from RCTs studies on efficacy of outpatient DBT as well as other effectiveness studies, our findings demonstrate improvement in symptoms for patients with borderline personality disorder over an initial phase of treatment followed by a 12-month treatment period in accordance with the German guidelines of psychotherapy. Our results indicate a reduction in the number of non-suicidal self-injury (NSSI), the number and duration of inpatient hospital stays, the severity of borderline symptoms as well as in depression and the global severity of symptoms (BSI). Only 23% of patients still met diagnostic criteria for BPD according to DSM-IV-TR after one year. It should be noted, however, that like in other studies the level of depression and general symptom severity improved but were still in a clinical range.

The study evaluated a therapy in a network of clinicians and institutions working with borderline patients in Berlin. The network was implemented in 2003 in order to improve the situation for borderline patients seeking treatment. The treatment approach of the network is DBT offered according to the guidelines formulated by Linehan [1]. Except for some of the inclusion- and exclusion criteria as well as patient referral, all other mentioned restrictions (e.g., patients were considered dropouts when they missed four or more consecutive scheduled sessions) are part of the DBT-rules within the Berlin borderline network.

In DBT the first five sessions define the initial phase of therapy. The main aim of this phase is getting a strong commitment for therapy from the patient. Our data show significant reduction of self-reported depressive symptoms (BDI) and borderline symptom severity (BSL) in the course of this initial phase (t0-t1) already. It should be noted that this initial phase included in some cases quite a long waiting time for an available therapist. Therefore, the knowledge about the availability of a therapy even after a long waiting period as well as further unknown variables may have played an additional role in this improvement.

Based on the multilevel analyses, further statements on changes in the course of therapy can be made for the severity of borderline symptoms as well as associated psychopathology. Significant short-term effects for the first four months of therapy (t2) were found only for BSI-GSI and the HAM-D. Over the complete 12 months (t1 to t3), significant improvement was obtained for nearly all self-reported measures assessing psychopathological symptom severity. The lack of improvement on the BSL over the one-year period is somewhat surprising. This may be due to the improvement in borderline symptom severity during the initial phase (t0-t1) already.

In addition to the mixed-model completer analyses, an ITT analysis with *n* =78 patients was conducted. Because the results for the ITT analysis did not differ noticeably from those of the completer analyses, it can be assumed that participant attrition did not substantially alter the pattern of findings.

In comparison to the study of Bohus et al. [47] significant change and reliable change rates are somewhat lower in our study. Bohus et al. reported that 41.9% of the BPD patients receiving 3 months of inpatient DBT were clinically recovered on a general measure of psychopathology [46]; in our study 38.5% of the participants receiving one-year outpatient therapy were clinically recovered or improved using the same self-report instrument (BSI-GSI [39]; on average across all applied instruments: 36.9%). Furthermore, our outcome shows a somewhat lower but still comparable rate of improvement for BPD symptoms and psychopathology compared to the treatment of avoidant personality disorder (e.g. [48]).

The overall attrition rate including the patients who prematurely terminated the study protocol but not the therapy was 32.9%, the rate for treatment dropout only was 24.3%. Thus the current dropout rate (32.9%) is higher than in most other studies evaluating DBT with BPD patients ([2–4], [6–8], [23–27]). Only in the two studies by McMain et al. [9] and Verheul et al. [5] the dropout rates were comparable, in the trial by Feigenbaum et al. [2] the dropout rate was even higher (56%). It should be noted that DBT drop-out rates are difficult to compare between studies due to different treatment duration and different definition of drop-out (for a discussion see Kröger et al. 2014 [49]). Unfortunately, the reasons for therapy or study discontinuation could only partially be explained (see Figure 1). Almost half of the 23 patients terminated their further participation without giving any reasons. However, we assume that the attrition rate of our study is representative of the health care situation in Germany. In another German sample Kroeger et al. [49] found nearly the same attrition rate within a 3-month DBT inpatient treatment.

In our study, the additional attendance of team consultations by therapists may have served as a protection against patients’ discontinuation of therapy. This finding confirms the statement by Linehan according to which therapists treating borderline patients need support on a regular basis. However, it remains unclear whether the consultation team attendance was decisive for the lower dropout rate or whether it was due to a higher motivation of therapists or other variables that were not assessed. In contrast to the results of Pasieczny & Conner [27], the extent of DBT training had no impact on treatment outcome. In our opinion further research should address the influence of these therapist-specific variables in a systematic manner.

To examine comparability to other studies, the severity of the disorder, the dose of therapy as well as the quality of the therapy procedure (adherence) of the different samples should be considered. To the extent to which the measurement instruments were comparable, on a descriptive level the severity of the disorder of the participants in the current study was similar to that reported in publications by Koons et al. [3], Hjalmarsson et al. [26], Linehan et al. [4], McMain et al. [9], Pasieczny & Conner [27] and Turner [7]. The sample examined by Friedrich et al. [25] seemed somewhat less severely impaired, the one examined by Linehan et al. [8] more severely affected. Regarding comorbidity with other Axis-I and/or Axis-II disorders, our sample is also comparable with those in aforementioned publications. Only the number of acute comorbid depressive disorders was slightly lower than the average number in the other studies [2, 8, 9, 24, 27]. This is also reflected in the comparably low value of HAM-D. The extent of therapy received was comparable to that reported in McMain et al. [9] (individual therapy sessions: 39 vs. 32; skills group 19 vs. 26). In comparison to the study by Linehan et al. [8] participants in the current study received slightly less treatment (individual therapy: 39 vs. 43; skills group: 19 vs. 38). Therapists’ adherence was good with a score of 4.17 (on a scale from 0-5) (Linehan et al. [8]: 4.0; McMain et al. [9]: 4.06). In summary, concerning the comparability with other treatment studies, we see no limitations of our outcome data regarding severity of the disorder, dose of therapy and adherence.

The pre-post effect sizes of the dependent variables were medium in size (following the interpretation of Cohen [45]). A small to median effect was found for NSSI; for the number of BPD criteria a large effect was found. To date, Comtois et al. [24] have been the sole research team to compare their outcomes with those of three RCTs [4, 8, 50], within a benchmarking design. In a descriptive comparison, the study by Comtois et al. [24] showed similarly good results as the RCTs, with the exception of the reduction of medically treated parasuicides (NSSI: *d* =0.13; inpatient-days: *d* =0.55). The effect sizes of our study, too, are comparable to those of other effectiveness and efficacy studies. However, regarding the reduction in NSSI our effect size is somewhat smaller [3, 9, 26, 27], but not as small as in the study of Comtois et al. [24].

### Limitations

A main limitation of our study is the large number of missing data that may have led to biased results. Limited financial as well as personnel resources may have discouraged study participants from regularly participating at the assessment procedures to the desired extent.

A further limitation is the absence of a control group. For this reason we cannot clearly attribute progress to the specific DBT interventions. For instance, our results demonstrate a decline in Borderline symptoms between t0 and t1 possibly due to the knowledge of a therapy slot with an expert in treating BPD, and the first therapy sessions. This interpretation could be strengthened by the comparison with a control group. However, it should be noted that our results are comparable to those in other effectiveness and efficacy studies.

The generalizability of the current results to all BPD patients may be somewhat limited because exclusion criteria were rather stringent to ensure comparability with other outcome trials of DBT. Patients with current substance dependence, acute suicidality, and psychotic symptoms are usually not treated in outpatient settings in Germany.

To this date, it is quite difficult for patients with BPD in Germany to find a therapist who is willing to offer psychotherapeutic treatment. Therefore, it can be assumed that a number of patients originally took part in the study in order to obtain a therapy slot. Some of them may have lost interest in the research setting once they had started their treatment and may thus have dropped out of the protocol. As the pre-assessment scores (t0) on the dependent variables did not differ from those of the completers, an impact of psychopathology on the dropout behavior can be ruled out.

## Conclusions

To summarize, by using outpatient DBT under routine health care conditions in Germany significant improvement of psychopathology in borderline patients was observed. In line with the results of other effectiveness studies by Comtois et al. [24], Friedrich et al. [25], Hjalmarsson et al. [26], and Pasieczny & Conner [27], our data suggest that DBT treatment in routine health care is efficient and its outcome is comparable to that of RCTs.

## References

[1] Linehan MM (1993). Cognitive-behavioral treatment of borderline personality disorder.

[2] Feigenbaum JD, Fonagy P, Pilling S, Jones A, Wildgoose A, Bebbington PE (2012). A real-world study of the effectiveness of DBT in the UK National Health Service. Br J Clin Psychol.

[3] Koons CR, Robins CJ, Tweed JL, Lynch TR, Gonzalez AM, Morse JQ, Bishop GK, Butterfield MI (2001). Efficacy of dialectical behavior therapy in women veterans with borderline personality disorder. Behav Ther.

[4] Linehan MM, Armstrong HE, Suarez A, Allmon D, Heard HL (1991). Cognitive-behavioral treatment of chronically parasuicidal borderline patients. Arch Gen Psychiatry.

[5] Verheul R, Van Den Bosch LM, Koeter MW, De Ridder MA, Stijnen T, Van den Brink W (2003). Dialectical behaviour therapy for women with borderline personality disorder: 12-month, randomised clinical trial in The Netherlands. Br J Psychiatry.

[6] Carter GL, Willcox CH, LEwin TJ, Conrad AM, Bendit N (2010). Hunter DBT project: randomized controlled trial of dialectical behaviour therapy in women with borderline persoanlity disorder. Aust N Z J Psychiatry.

[7] Turner RM (2000). Naturalistic evaluation of dialectical behavior therapy-oriented treatment for borderline personality disorder. Cogn Behav Pract.

[8] Linehan MM, Comtois KA, Murray AM, Brown MZ, Gallop RJ, Heard HL, Korslund KE, Tutek DA, Reynolds SK, Lindenboim N (2006). Two-year randomized controlled trial and follow-up of dialectical behavior therapy vs therapy by experts for suicidal behaviors and borderline personality disorder. Arch Gen Psychiatry.

[9] McMain SF, Links PS, Gnam WH, Guimond T, Cardish RJ, Korman L, Streiner DL (2009). A randomized trial of dialectical behavior therapy versus general psychiatric management for borderline personality disorder. Am J Psychiatry.

[10] Clarkin JF, Levy KN, Lenzenweger MF, Kernberg OF (2007). Evaluating three treatments for borderline personality disorder: a multiwave study. Am J Psychiatry.

[11] Stoffers JM, Vollm BA, Rucker G, Timmer A, Huband N, Lieb K (2012). Psychological therapies for people with borderline personality disorder. Cochrane Database Syst Rev.

[12] DeutscheGesellschaftFürPsychiatriePsychotherapieUndNervenheilkunde (2009). Leitlinien für Persönlichkeitsstörungen.

[13] AmericanPsychologicalAssociation (2014). Society of Clinical Psychology.

[14] Linehan MM, Heard HL, Miller N, Magruder KM (1999). Borderline personality disorder: costs, course, and treatment outcomes. The Cost-Effectiveness of Psychotherapy: A Guide for Practitioners, Researchers and Policy-Makers.

[15] Priebe S, Bhatti N, Barnicot K, Bremner S, Gaglia A, Katsakou C, Molosankwe I, McCrone P, Zinkler M (2012). Effectiveness and cost-effectiveness of dialectical behaviour therapy for self-harming patients with personality disorder: a pragmatic randomised controlled trial. Psychother Psychosom.

[16] Wagner T, Fydrich T, Stiglmayr C, Marschall P, Salize H-J, Renneberg B, Fleßa S, Roepke S (2014). Societal cost-of-illness in patients with borderline personality disorder one year before, during and after dialectical behaviour therapy in routine outpatient care. Behav Res Ther.

[17] Wagner T, Roepke S, Marschall P, Stiglmayr C, Renneberg B, Gieb D, Dambacher C, Matthies S, Salbach-Andrae H, Fleßa S, Fydrich T (2013). Krankheitskosten der Borderline Persönlichkeitsstörung aus gesellschaftlicher Perspektive. Z Klin Psychol Psychother.

[18] Rounsaville BJ, Carroll KM, Onken LS (2001). A Stage Model of Behavioral Therapies research: getting started and moving on from stage I. Clinical Psychology-Science and Practice.

[19] Binks CA, Fenton M, McCarthy L, Lee T, Adams CE, Duggan C (2006). Psychological therapies for people with borderline personality disorder. Cochrane Database Syst Rev.

[20] Paris J (2008). Treatment of borderline personality disorder: a guide to evidence-based practice.

[21] Swenson CR, Torrey WC, Koerner K (2002). Implementing dialectical behavior therapy. Psychiatr Serv.

[22] National Collaborating Centre For Mental Health (2009). Borderline personality disorder: the NICE Guideline on Treatment and management.

[23] Brassington J, Krawitz R (2006). Australasian dialectical behaviour therapy pilot outcome study: effectiveness, utility and feasibility. Australas Psychiatry.

[24] Comtois KA, Elwood L, Holdcraft LC, Smith WR, Simpson TL (2007). Effectiveness of dialectical behavior therapy in a community mental health center. Cogn Behav Pract.

[25] Friedrich J, Gunia H, Huppertz M (2003). Evaluation eines ambulanten Netzwerks für Dialektisch Behaviorale Therapie. Verhaltenstherapie & Verhaltensmedizin.

[26] Hjalmarsson E, Kåver A, Perseius K-I, Cederberg K, Ghaderi A (2008). Dialectical behaviour therapy for borderline personality disorder among adolescents and young adults: Pilot study, extending the research findings in new settings and cultures. Clinical Psychologist.

[27] Pasieczny N, Connor J (2011). The effectiveness of dialectical behaviour therapy in routine public mental health settings: An Australian controlled trial. Behav Res Ther.

[28] Jobst A, Hörz S, Birkhofer A, Martius P, Rentrop M (2010). Einstellung von Psychotherapeuten gegenüber der Behandlung von Patienten mit Borderline Persönlichkeitsstörung. Psychother Psychosom Med Psychol.

[29] Menninger WW (1991). Patient suicide and its impact on the psychotherapist. Bull Menninger Clin.

[30] Skodol AE, Buckley P, Charles E (1983). Is there a characteristic pattern to the treatment history of clinic outpatients with borderline personality. J Nerv Ment Dis.

[31] AmericanPsychiatricAssociation (2000). Diagnostic and Statistical Manual of Mental Disorders.

[32] Fydrich T, Renneberg B, Schmitz B, Wittchen HU (1997). SKID-II. Strukturiertes Klinisches Interview für DSM-IV. Achse II: Persönlichkeitsstörungen.

[33] Wittchen H-U, Wunderlich U, Gruschwitz S, Zaudig M (1997). SKID-I. Strukturiertes Klinisches Interview für DSM-IV, Achse I: Psychische Störungen.

[34] Schmidt K-H, Metzler P (1992). Wortschatztest (WST).

[35] Linehan MM, Comtois KA (1994). Lifetime Parasuicide Count (LPC).

[36] Bohus M, Limberger MF, Frank U, Sender I, Gratwohl T, Stieglitz RD (2001). Development of the Borderline Symptom List. Psychother Psychosom Med Psychol.

[37] Renneberg B, Schmidt-Rathjens C, Hippin R, Backenstrass M, Fydrich T (2005). Cognitive characteristics of patients with borderline personality disorder: development and validation of a self-report inventory. J Behav Ther Exp Psychiatry.

[38] Renneberg B, Seehausen A (2010). Fragebogen zu Gedanken und Gefühlen (FGG): Ein Screening Instrument für Borderline-spezifisches Denken. Zeitschrift für Klinische Psychologie und Psychotherpie.

[39] Franke GH (2000). BSI: Brief Symptom Inventory – Deutsche Version Manual.

[40] Hautzinger M, Bailer M, Worrall H, Keller F (2000). Beck-Depressions-Inventar (BDI). Testhandbuch.

[41] CollegiumInternationalePsychiatriaeScalarum (2005). Internationale Skalen für Psychiatrie.

[42] Stiglmayr C, Schimke P, Wagner T, Braakmann D, Schweiger U, Sipos V, Fydrich T, Schmahl C, Ebner-Priemer U, Kleindienst N, Bischkopf J, Auckenthaler A, Kienast T (2010). Development and psychometric characteristics of the Dissociation Tension Scale. J Pers Assess.

[43] Linehan MM, Korslund KE (2003). Dialectical Behavior Therapy Adherence Manual.

[44] Barr DJ, Levy R, Scheepers C, Tily HJ (2013). Random effects structure for confirmatory hypothesis testing: keep it maximal. J Mem Lang.

[45] Cohen J (1988). Statistical power analysis for the behavioral sciences.

[46] Jacobson NS, Roberts LJ, Berns SB, McGlinchey JB (1999). Methods for defining and determining the clinical significance of treatment effects: description, application, and alternatives. J Consult Clin Psychol.

[47] Bohus M, Haaf B, Simms T, Limberger MF, Schmahl C, Unckel C, Lieb K, Linehan MM (2004). Effectiveness of inpatient dialectical behavioral therapy for borderline personality disorder: a controlled trial. Behav Res Ther.

[48] Renneberg B, Goldstein AJ, Phillips D, Chambless DL (1990). Intensive behavioral group treatment of avoidant personality-disorder. Behav Ther.

[49] Kroeger C, Roepke S, Kliem S (2014). Reasons for premature termination of dialectical behavior therapy for inpatients with borderline persoanlity disorder. Behav Res Ther.

[50] van den Bosch LM, Koeter MW, Stijnen T, Verheul R, van den Brink W (2005). Sustained efficacy of dialectical behaviour therapy for borderline personality disorder. Behav Res Ther.

